# 

**Published:** 1882-02

**Authors:** 


### CONTENTS:

PAGE.

ORIGINAL COMMUNICATIONS:

Cephalic Version in the Postural Position. By John Hauenstein, M. D., . . . 289

CLINICAL REPORTS:

Cerebral Tumor involving the Nerves of the Special Senses. By Lucien Howe, M.D., 299
Notes from Practice—Obstruction of the Bowels, with History and Autopsy of Three Cases. Reported by Thomas Lothrop, M. D., . . . . . . 302

SELECTIONS:

Vaginal Chancres, . . . . . . . 310
Iodoform as a Dressing for Wounds, . . 312
Obstinate Vomiting in Pregnancy, . . 313
Inunction of Sapo Viridis in Scrofula, . . 315
Hypertrophy and Dilatation of the Heart during Scarlet-Fever Nephritis, . . 316
Treatment of Acneiform Rash due to Bromide of Potassium, while continuing the Remedy, . . . . . . . . 317
Sulphide of Calcium for Suppurating Buboes, 317

PAGE.

Nascent Iodide of Silver in the Treatment of Conjunctivitis, . . . . . . . 318
Chlorate of Potash Poisoning, . . . 319
Pyrogallic Acid in Phagædenic Ulceration, 321
Note on a New Antipruritic Remedy, . . 321
Soothing Ointments and the Indications for their Use, . . . . . . . 321
Local Mercurial Fumigation, . . . 322
Fœtid Sweating of the Feet, . . . . 322
Vaccination a Relic of Barbarism, . . 323
Rapid Cure of Lichen Ruber by Hypodermic Injections of Arsenic, . . . . 323
"The Sun do Move."—Anti-Vaccinationists, 324

SOCIETY REPORTS:

Annual Meeting of the Erie County Medical Society, . . . . . . . . 326

EDITORIAL:

The Board of Health, . . . . . . 329
"This, this is Bliss," . . . . . . 331
Editorial Notes, . . . . . . . 332

REVIEWS, . . . . . . . . 334

